# Fatigue and recovery have different effects on knee biomechanics of drop vertical jump between female collegiate and recreational athletes

**DOI:** 10.1186/s13018-021-02893-6

**Published:** 2021-12-30

**Authors:** Kengo Harato, Yutaro Morishige, Yasuo Niki, Shu Kobayashi, Takeo Nagura

**Affiliations:** 1 Department of Orthopaedic Surgery, Keio University School of Medicine, 35 Shinanomachi, Shinjukuku, Tokyo 160-8582 Japan; 2 Department of Clinical Biomechanics, Keio University School of Medicine, 35 Shinanomachi, Shinjukuku, Tokyo 160-8582 Japan

**Keywords:** Landing task, Activity level, Motion capture system, Three-dimensional assessment, Knee abduction moment

## Abstract

**Background:**

Although fatigue is known as one of the risk factors for noncontact anterior cruciate ligament injury, the effects of fatigue and recovery can be different based on the level of competition. However, it is unknown whether female recreational athletes are susceptible to fatigue or not, compared to female collegiate athletes with greater physical activity. The purpose of the present study was to examine and clarify the effects of fatigue and recovery on knee biomechanics of the drop vertical jump (DVJ) in female recreational athletes compared to female collegiate athletes.

**Methods:**

Fifteen female collegiate athletes and ten female recreational athletes were enrolled in the current study. All subjects were basketball players and Tegner activity scales were level 9 and 7, respectively. They performed DVJ before and after the fatigue protocol. Three-dimensional knee kinematics and kinetics were collected during landing phase of DVJ. The data after the fatigue protocol (first, second, and third DVJs) were compared with those before the protocol using one-way repeated measures of analysis of variance in each group.

**Results:**

Fatigue caused significant increase of knee abduction angle at initial contact (IC) and peak abduction moments within 40 ms from IC in female recreational athletes, whereas no increases of these parameters were observed in female collegiate athletes. Moreover, recovery from fatigue seemed to be more slowly in female recreational athletes than in female collegiate athletes as smaller knee flexion moment was observed even in post-fatigue third DVJ only for female recreational athletes.

**Conclusions:**

Effects of fatigue on DVJ were significantly greater and continued for a longer duration in female recreational athletes compared to female collegiate athletes.

## Introduction

The anterior cruciate ligament (ACL) injury is a common sports-related trauma in young athletes, and approximately 200,000 tears occur each year in the USA [1]. The injury mechanism has been a highly disputed topic, as 70 to 90% of ACL tears are related to noncontact injuries such as landing from a jump, a quick stop, or a change of direction (cutting maneuver) [2, 3]. Recently, motion capture system has been used to assess the biomechanics of the injury mechanism. For instance, the drop vertical jump (DVJ) has been done to evaluate risky movements for ACL injury during landing from a jump [4, 5]. Hewett et al. conducted a prospective cohort study and concluded that females who displayed increased knee valgus angle and increased external knee valgus moments during a drop-landing task were at an increased risk of sustaining a noncontact ACL injury [4]. Besides, although laboratory-based motion analysis systems are undoubtedly the gold standard, the Landing Error Scoring System (LESS) is an inexpensive clinical assessment tool using two standard video cameras to evaluate landing biomechanics. Padua et al. concluded that the LESS would be a valid and reliable tool for identifying potentially high-risk movement patterns during a jump-landing task [6].

According to previous studies, fatigue could be one of the risk factors for noncontact ACL injury [7–15]. Haddas et al. reported that females exhibited biomechanical factors that might increase their risk of ACL injury during landing from a 0.30-m height when compared with males, particularly when landing after fatigue [9]. In their study, delays in semitendinosus, multifidus, gluteus maximus, and rectus femoris activation were observed after fatigue. A delay in knee-muscle activity has been suggested to be a major risk factor for knee instability and ACL injury risk [11]. However, their subjects had recurrent low back pain and their activity level was unknown. Mejane et al. investigated the biomechanical study during landing tasks of female recreational athletes and indicated that neuromuscular fatigue could alter knee kinematics of landing with potential increase of noncontact ACL injury risk [14]. These reports are consistent with observations that most of noncontact ACL injuries occur at the end of a half or end of a game, when fatigue is highest [16, 17]. On the other hand, recovery from the fatigue is also a key consideration for athletes [18–20]. Tsai et al. assessed knee mechanics during side-step cutting immediately after a fatigue protocol and after 20 and 40 min of rest in female recreational athletes [19]. They indicated that fatigue resulted in changes in knee mechanics that could be associated with ACL injury, and suggested that forty minutes of recovery was not sufficient in restoring knee mechanics to pre-fatigue levels. The intensity of physical activity or skill level is one of the important factors when considering the effect of fatigue and recovery on knee biomechanics. However, it is unknown whether female recreational athletes are susceptible to fatigue or not, compared to female collegiate athletes with greater physical activity. The purpose of the present study was to examine and clarify the effects of fatigue and recovery on the knee biomechanics of the DVJ in female recreational athletes compared to female collegiate athletes. It was hypothesized that knee kinematics, kinetics, and the LESS score in female recreational athletes would be deteriorated by fatigue compared to those in female collegiate athletes. Furthermore, effects of fatigue on DVJ could continue for a longer duration in female recreational athletes compared to female collegiate athletes.

## Methods

### Participants

A total of 25 female athletes were enrolled in the present study, and all subjects were basketball players. Fifteen female collegiate athletes (mean age = 20.0 ± 1.5 years, mean body mass index [BMI] = 21.5 ± 0.9 kg/m^2^) and ten female recreational athletes (mean age = 20.9 ± 1.2 years, BMI = 20.6 ± 1.5 kg/m^2^) participated. Collegiate athletes were members of competitive team in our whole university. On the other hand, recreational athletes were members of non-competitive team in our university school of medicine. Tegner activity scores among the collegiate and recreational athletes were 9 and 7, respectively. Based on practice schedule, we observed the actual practice and confirmed that physical demands in collegiate athletes were 3 h a day, five times a week, and those in recreational athletes were 3 h a day, three times a week, respectively. None of the subjects had any history of major injuries to the trunk and lower extremities. As female athletes are at greater risk of noncontact ACL injury than male athletes in noncontact sports [21, 22], females were chosen in the current study. An informed consent form approved by the institutional review board at our university (#20080054) was signed by each subject.

### Test procedures

The subjects performed double-legged drop landing and executed a vertical jump after landing (DVJ). Drop landing tasks were jumping from a 30-cm high box to a distance of 50% of their height away from the box onto force plates and immediately rebounding for a maximal vertical jump on landing.

Before data collection, subjects received instructions about performing the DVJ. DVJs in each subject were captured using a motion analysis system which consisted of eight cameras (120 frames/s; Oqus, Qualisys, Gothenburg, Sweden) and two force plates (frequency 600 Hz; AM6110, Bertec, Columbus, OH). The force plate collected ground reaction force (GRF) data synchronized to the camera sampling rate. GRF data were used to identify the time at initial contact (IC) and at toe-off from the jump (TO). Forty-six retroreflective markers (14 mm in diameter) were placed at the standard anatomical landmarks. Three non-collinear infrared markers were used to track each of the following 8 segments: 2 feet, 2 legs, 2 thighs, pelvis, and trunk. To define the axes of each of the 8 segments, an anatomical model was created by digitizing standard bony landmarks: bilateral acromion processes, the xiphoid process, the suprasternal notch, the seventh cervical vertebra, the tenth thoracic vertebra, bilateral anterior and posterior iliac spines, bilateral iliac crests, bilateral greater trochanters, bilateral lateral and medial epicondyles, bilateral lateral and medial malleoli, bilateral posterior heels, bilateral medial cuneiforms, bilateral great toes, and bilateral heads of the fifth metatarsals. Additional tracking markers were placed on the frontal aspects of the thigh (4 markers) and shank (4 markers). Calibration markers (bilateral medial epicondyles and medial malleoli) were removed after the standing trial, and only tracking markers were left on the subject throughout the entire data collection session.

### Fatigue protocol (FP)

**Fig. 1:**
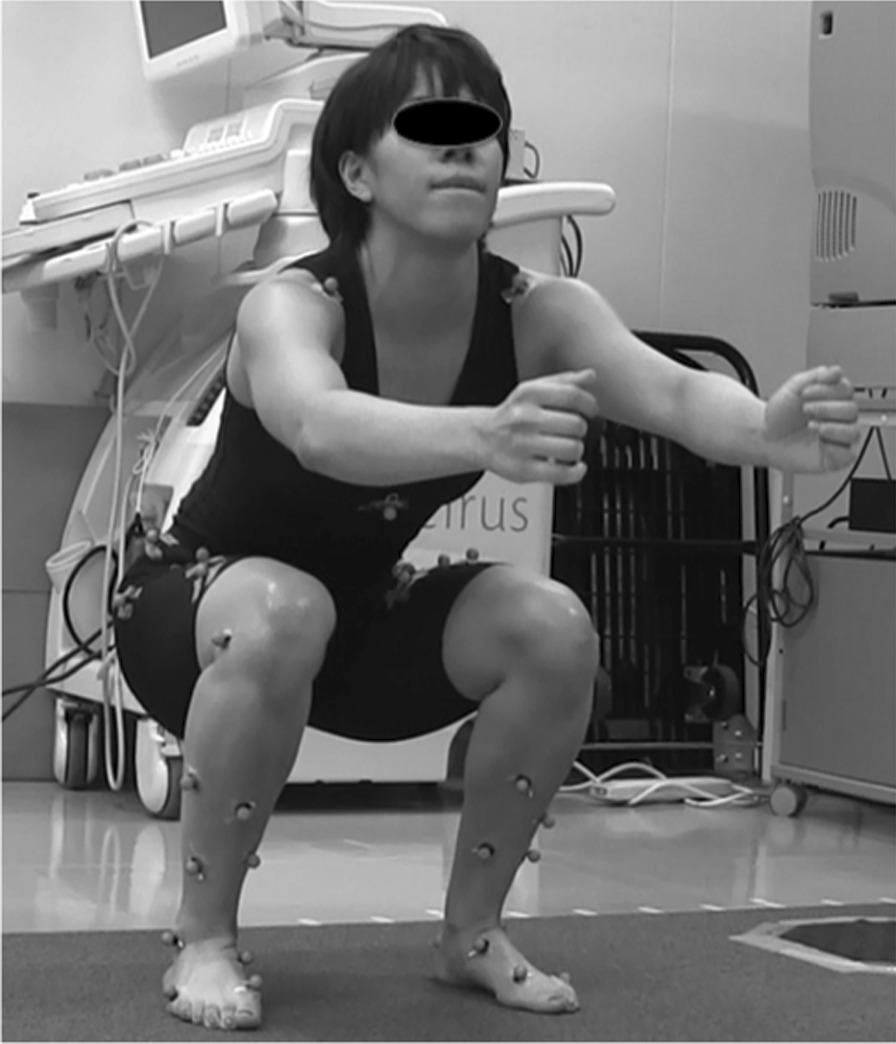
The fatigue protocol consisted of the subject performing double-legged squats, with arms parallel to the ground, to a depth of 90° knee flexion. The fatigue was operationally defined as the point when the participants could not accomplish the fatigue protocol

Subject performed double-legged squats, with arms parallel to the ground, to a depth of 90° knee flexion as the fatigue protocol (Fig. 1). The fatigue was operationally defined as the point when the subjects could not accomplish the FP. In addition, the rating of perceived exertion (RPE) score was checked to determine their level of fatigue using RPE sheet [23]. The RPE score is used as indicator reflecting the activity intensity. The goal of RPE score was set at greater than 17 in which subjects felt very hard.

After performing the DVJ several times as warm-ups, three trials were recorded on both limbs for each subject before the FP. Thereafter, subjects performed the post-FP first DVJ within 30 s. The post-FP second and third DVJs were done after every 90-s interval.

### Data assessment

For each subject, three-dimensional kinematic, kinetic, and GRF data were recorded bilaterally during IC to TO as the landing phase. The third trial before the FP and the three trials after the FP were analyzed. The motion of markers was recorded using Track Manager version 2.7 software (Qualisys). To calculate knee kinematics and kinetics, Visual 3D (C-motion Company, Rockville, MD) was used. The knee flexion angle at IC, the peak knee flexion angle (IC-TO), the knee abduction angle at IC, the peak knee abduction angle (IC-TO), the knee internal rotation angle at IC, and the peak knee internal rotation angle (IC-TO) were evaluated as kinematic parameters. Knee internal rotation was defined as tibial internal rotation with respect to the femur. In addition, as kinetic parameters, peak knee flexion moment, peak abduction moment, and peak internal rotation moment within 40 ms from IC were also evaluated [24]. Those moments were calculated as “external moment.” Simultaneously, frontal and sagittal view video data were acquired using standard HD video cameras (120 frames/s; Oqus, Qualisys) to evaluate the LESS.

### Statistical analysis

Two-tailed unpaired *t*-test or Mann–Whitney U-test was performed between female collegiate and recreational athletes after confirming normality assumption using the Shapiro–Wilk test. Subject demographics, squat times, pre-FP biomechanical data on dominant side were compared between female recreational and collegiate athletes. The dominant leg was determined by asking which leg they prefer to kick a ball [25]. Moreover, the data after the FP (first, second, and third) were compared with those before the FP using one-way repeated measures of analysis of variance in each group. After a significant *P* value was determined, a post hoc Bonferroni correction was performed to compare selected mean values. *P* value less than 0.05 was also considered as significant. All statistical analyses were done with the Microsoft Excel Statistical Package, version 2015 (Social Survey Research Information, Tokyo). A power analysis was performed using G*Power (v3.1.9.2, Heinrich-Heine University, Düsseldorf, Germany). Effect size (d) was calculated for the peak knee abduction moment within 40 ms before the FP in both groups. Thereafter, using a large effect size of 1.3 for two-tailed *t*-test, a sample size of 9 and 13 for each group was required (*β* = 0.80, *α* = 0.05). Thus, 10 and 15 participants were used in the present study.

## Results

The demographic data of the subjects are shown in Table 1. Age and BMI were not significantly different between the two groups. There were also no significant differences in the number of squats between the groups (*P* = 0.14), though there were 32% more squats performed by collegiate group. However, significant difference was detected in the average of basketball experience (9.8 ± 3.7 years in female collegiate athletes and 6.5 ± 4.4 years in female recreational athletes, *P* = 0.041).

**Table 1:** Subjects’ demographics (mean ± SD)

	Collegiate athletes (*N* = 15)	Recreational athletes (*N* = 10)	*P* value^a^
Age (yrs)	20.0 ± 1.5	20.9 ± 1.2	0.12
Body mass index (kg/m^2^)	21.5 ± 0.9	20.6 ± 1.5	0.07
Tegner activity score	9	7	
Experience (yrs)	9.8 ± 3.7	6.5 ± 4.4	0.041
Squat (times)	305.7 ± 134.7	229.8 ± 100.3	0.14

^a^Values obtained using two-tailed unpaired *t*-test or Mann–Whitney U-test

Among female collegiate athletes, the LESS score was significantly worse in post-FP first (5.7 ± 1.6), second (5.7 ± 1.6), and third (5.3 ± 1.8) DVJs compared to pre-FP DVJ (4.7 ± 1.3). In addition, significant differences were observed in peak knee flexion angle (pre-FP vs. post-FP first and second DVJs), while other kinematic and kinetic parameters were not different (Table 2).

**Table 2:** Kinematic and kinetic differences in female collegiate athletes (mean ± SD)

	Pre-FP DVJ	Post-FP first DVJ	Post-FP second DVJ	Post-FP third DVJ	*P* value^a^
LESS score	4.7 ± 1.3	5.7 ± 1.6*	5.7 ± 1.6**	5.3 ± 1.8***	< 0.001
Knee flexion angle at IC (Deg.)	31.7 ± 9.0	33.0 ± 10.6	29.8 ± 12.5	29.0 ± 10.3	0.127
Peak knee flexion angle (Deg.)	101.7 ± 13.0	108.0 ± 11.8*	106.3 ± 12.3**	101.6 ± 11.7	< 0.01
Knee abduction angle at IC (Deg.)	0.7 ± 8.1	0.4 ± 10.6	0.4 ± 10.0	− 0.1 ± 9.6	0.949
Peak knee abduction angle (Deg.)	9.1 ± 11.3	9.1 ± 13.2	9.7 ± 13.0	8.4 ± 12.0	0.843
Tibial internal rotation angle at IC (Deg.)	− 3.6 ± 9.0	− 4.2 ± 9.6	− 5.7 ± 10.6	− 5.2 ± 10.0	0.293
Peak tibial internal rotation angle (Deg.)	4.2 ± 6.1	5.7 ± 7.8	4.6 ± 8.3	5.0 ± 8.2	0.585
Peak knee flexion moment within 40 ms from IC (Nm/kg)	1.71 ± 0.38	1.63 ± 0.56	1.40 ± 1.05	1.60 ± 0.50	0.639
Peak knee abduction moment within 40 ms from IC (Nm/kg)	0.53 ± 0.23	0.48 ± 0.37	0.49 ± 0.26	0.50 ± 0.29	0.918
Peak tibial internal rotation moment within 40 ms from IC (Nm/kg)	0.14 ± 0.15	0.17 ± 0.17	0.13 ± 0.10	0.11 ± 0.13	0.618

*IC* initial contact, *FP* fatigue protocol, *ms* milliseconds, *NS* not significant

*P < 0.05 between pre- and post-FP first DVJ

**P < 0.05 between pre- and post-FP second DVJ

***P < 0.05 between pre- and post-FP third DVJ

^a^Values obtained using repeated measures of analysis of variance among groups

Among female recreational athletes, similarly, the LESS score was significantly worse in post-FP first (6.2 ± 2.3), second (6.2 ± 2.1), and third (5.9 ± 2.2) DVJs compared to pre-FP DVJ (5.0 ± 1.7) (Table 3). Moreover, significant differences were observed in peak knee flexion angle (pre-FP vs. post-FP first, second, and third DVJs) and in knee abduction angle at IC (pre-FP vs. post-FP first and second DVJs). In terms of kinetics, peak knee flexion moment within 40 ms from IC was significantly smaller (pre-FP vs. post-FP second and third DVJs), and peak knee abduction moments within 40 ms from IC were significantly greater in post-FP first DVJ than in pre-FP DVJ.

**Table 3:** Kinematic and kinetic differences in female recreational athletes (mean ± SD)

	Pre-FP DVJ	Post-FP first DVJ	Post-FP second DVJ	Post-FP third DVJ	*P* value^a^
LESS Score	5.0 ± 1.7	6.2 ± 2.3*	6.2 ± 2.1**	5.9 ± 2.2***	0.034
Knee flexion angle at IC (Deg.)	35.0 ± 7.3	33.5 ± 6.9	30.4 ± 6.2	32.3 ± 6.3	0.330
Peak knee flexion angle (Deg.)	95.9 ± 11.0	101.6 ± 12.4*	108.2 ± 14.9**	104.2 ± 12.5***	< 0.01
Knee abduction angle at IC (Deg.)	− 3.7 ± 4.4	− 2.0 ± 5.3*	− 2.2 ± 3.6**	− 3.2 ± 4.8	0.047
Peak knee abduction angle (Deg.)	2.4 ± 5.7	2.7 ± 4.8	2.6 ± 3.8	3.3 ± 6.0	0.833
Tibial internal rotation angle at IC (Deg.)	− 10.8 ± 11.0	− 9.6 ± 10.9	− 11.4 ± 12.6	− 10.7 ± 12.0	0.365
Peak tibial internal rotation angle (Deg.)	− 0.39 ± 8.0	1.5 ± 8.5	0.2 ± 9.1	− 1.1 ± 8.9	0.122
Peak knee flexion moment within 40 ms from IC (Nm/kg)	1.78 ± 0.55	1.54 ± 0.45	1.26 ± 0.52**	1.31 ± 0.31***	< 0.01
Peak knee abduction moment within 40 ms from IC (Nm/kg)	0.21 ± 0.25	0.41 ± 0.24*	0.21 ± 0.17	0.23 ± 0.14	0.017
Peak tibial internal rotation moment within 40 ms from IC (Nm/kg)	0.25 ± 0.17	0.19 ± 0.11	0.28 ± 0.19	0.20 ± 0.18	0.460

*IC* initial contact, *FP* fatigue protocol, *ms* milliseconds, *NS* not significant

*P < 0.05 between pre- and post-FP first DVJ

**P < 0.05 between pre- and post-FP second DVJ

***P < 0.05 between pre- and post-FP third DVJ

^a^Values obtained using repeated measures of analysis of variance among groups

Concerning the comparison between groups, peak knee abduction moment within 40 ms from IC before the FP was significantly larger in female collegiate athletes than in female recreational athletes (Table 4).

**Table 4:** Kinematic and kinetic differences between female collegiate and recreational athletes before the fatigue protocol (mean ± SD)

	Pre-FP Female collegiate athletes	Pre-FP Female recreational athletes	*P* value^a^
LESS Score	4.7 ± 1.3	5.0 ± 1.7	0.665
Knee flexion angle at IC (Deg.)	31.7 ± 9.0	35.0 ± 7.3	0.338
Peak knee flexion angle (Deg.)	101.7 ± 13.0	95.9 ± 11.0	0.256
Knee abduction angle at IC (Deg.)	0.7 ± 8.1	− 3.7 ± 4.4	0.130
Peak knee abduction angle (Deg.)	9.1 ± 11.3	2.4 ± 5.7	0.097
Tibial internal rotation angle at IC (Deg.)	− 3.6 ± 9.0	− 10.8 ± 11.0	0.081
Peak tibial internal rotation angle (Deg.)	4.2 ± 6.1	− 0.39. ± 8.0	0.122
Peak knee flexion moment within 40 ms from IC (Nm/kg)	1.71 ± 0.38	1.78 ± 0.55	0.718
Peak knee abduction moment within 40 ms from IC (Nm/kg)	0.53 ± 0.23	0.21 ± 0.25	< 0.01
Peak tibial internal rotation moment within 40 ms from IC (Nm/kg)	0.14 ± 0.15	0.25 ± 0.17	0.101

*IC* initial contact, *FP* fatigue protocol, *ms* milliseconds, *ns* not significant

^a^Values obtained using two-tailed unpaired *t*-test

## Discussion

The results of the present study partly supported our hypothesis that knee kinematics and kinetics in female recreational athletes would be more affected by fatigue than those in female collegiate athletes, even if the LESS score was significantly worse in post-FP DVJs, compared to pre-FP DVJ in both groups. Moreover, recovery time from fatigue seemed to be longer in female recreational athletes than in female collegiate athletes as smaller knee flexion moment reflected the knee extensor fatigue. Specifically, peak knee flexion angle and peak knee flexion moment within 40 ms after IC did not return to pre-FP level in female recreational athletes. The most important finding of the current investigation was that fatigue caused significant increase of the knee abduction angle at IC and peak abduction moments within 40 ms from IC in female recreational athletes, whereas no increases of these parameters were observed in female collegiate athletes. Therefore, this study suggests that fatigue has different effects between female collegiate and recreational athletes. However, larger peak knee abduction moment within 40 ms after IC were observed in female collegiate athletes, compared to female recreational athletes before the FP. Presumably, this might have been a selection bias.

According to previous biomechanical studies, frontal-plane knee mechanics have prospectively predicted ACL injury, with greater abduction linked to ligament rupture [2, 5, 26]. When knee valgus is applied, the amount of the ACL load is greatly magnified. Thus, greater knee abduction angle and moment during landing have been thought to be important biomechanical risk factors for ACL injury. The ACL injury mechanism was investigated using video sequences from women’s handball and basketball in a previous report [2]. A model-based image-matching method was used, and rapid valgus development within 40 ms after IC was seen in the timing of the ACL injury. Therefore, valgus loading could be a key factor in the ACL injury mechanism. In the present study, significant increases of the knee abduction angle at IC and peak abduction moments within 40 ms from IC during post-FP first DVJ were observed in female recreational athletes, while no increases of these parameters were seen in female collegiate athletes. Therefore, our findings suggest that fatigue may have a larger impact on the risk of ACL injury in recreational versus collegiate female athletes.

In terms of fatigue, several protocols have been reported, including repeating vertical jump, uphill treadmill walking, actual football match, running, and squatting [7–15]. Despite the fact that some reports used a fatigue protocol only for the quadriceps muscle or hamstring muscle, the fatigue protocol in the current study was squatting, since the main focus was to create a situation that simulates the fatigue seen in the actual athletic activities. RPE is widely used to confirm the fatigue status [27, 28]. The aim of the RPE is to minimize the error and to maximize the accuracy of the fatigue protocol. An RPE of greater than 17 is a good indicator for the appropriate fatigue status. Recovery from the fatigue is also an important topic for athletes. Although fatigue may be associated with the increased risk of noncontact ACL injury [8, 13], little attention has been paid to the time required to restore normal knee kinematics and kinetics to pre-fatigue level. Most studies have focused on the recovery of muscle strength as opposed to the recovery of lower extremity mechanics [29–32]. As described, Tsai et al. assessed knee mechanics during side-step cutting in female recreational athletes and suggested that forty minutes of recovery was not sufficient in restoring knee mechanics to pre-fatigue levels [19]. In the present study, post-FP third DVJ (180 s after squatting) seemed to restore normal knee kinematics and kinetics to pre-fatigue level only in female collegiate athletes. Therefore, recovery from fatigue as well as the effect of fatigue is different between groups.

Several limitations should be noted in the present study. First, it is not known whether the difference that was observed here between female collegiate and recreational athletes, and also the pre- and post-fatigue differences, would translate into an actual higher risk in ACL injury, since the exact threshold that increases the risk is not established. Second, electromyography data could not be measured in the present study, because many markers were placed to obtain accurate kinematic and kinetic data. Specific muscle contraction before and after the FP was thus not evaluated, though weakness in the quadriceps and hamstring muscles should be observed after squatting. Third, the LESS score was significantly worse in both groups after the FP. In the present study, kinematic and kinetic data were obtained only at the knee joint. Therefore, hip, ankle, pelvis, and trunk movements were not investigated. Forth, skin or soft tissue motion error of DVJ is unknown in our laboratory as Visual 3D is a reliable tool. Lastly, the actual incidence of ACL injuries in both groups could not be assessed. However, the results of the current study provide important information when considering the effects of fatigue and recovery on the knee biomechanics in female collegiate and recreational athletes.

## Conclusions

Knee biomechanics during DVJ after fatigue were notably different between female recreational and collegiate athletes. Specifically, significant increase of the knee abduction angle at IC and peak abduction moments within 40 ms from IC during DVJ after fatigue were observed in female recreational athletes. In addition, recovery time from fatigue seemed to be longer in female recreational athletes than in female collegiate athletes as knee extensor fatigue was still observed during post-fatigue third DVJ only in female recreational athletes. Effects of fatigue on DVJ in female recreational athletes were significantly greater and continued for a longer duration compared to female collegiate athletes.

## Data Availability

All supporting data can be provided based on request to the authors.

## Abbreviations

ACL: Anterior cruciate ligament
DVJ: Drop vertical jump
LESS: Landing Error Scoring System
BMI: Body mass index
GRF: Ground reaction force
IC: Initial contact
TO: Toe off
FP: Fatigue protocol
RPE: Rating of perceived exertion
